# Temperature-Dependent Development of the Post-Diapause Periods of the Apricot Seed Wasp *Eurytoma maslovskii* (Hymenoptera: Eurytomidae): An Implication for Spring Emergence Prediction Models

**DOI:** 10.3390/insects13080722

**Published:** 2022-08-12

**Authors:** Hai Nam Nguyen, In Jun Lee, Hyuck Joo Kim, Ki-Jeong Hong

**Affiliations:** 1 Department of Plant Medicine, Sunchon National University, Suncheon 57922, Korea; 2 Plant Protection Research Institute, Vietnam Academy of Agricultural Sciences, Hanoi 11910, Vietnam; 3 Department of Divergent Biosystems Engineering, Sunchon National University, Suncheon 57922, Korea

**Keywords:** cumulative flight, degree day, development rate, lower threshold, model selection, prolonged diapause

## Abstract

**Simple Summary:**

*Eurytoma maslovskii* is an important stone fruit pest whose control relies on application timing, which requires a reliable tool for predicting spring emergence. The most common approach in insect phenological modeling is based on temperature-dependent development and thermal bioparameters. However, not a lot is known regarding *E. maslovskii*. In the present study, we investigate the development of *E. maslovskii* after diapause breaks at various temperatures. The lower temperature threshold and thermal constant of the species after diapause are estimated. The results confirm that the field emergence of *E. maslovskii* can be predicted by a model using accumulated degree days starting from 1 January, thereby providing an efficient tool for pest-control decision making.

**Abstract:**

The present study investigates the influence of temperature on the development of *Eurytoma maslovskii* after a diapause break up until adulthood. The insect development rate was fitted to both linear and nonlinear models to estimate thermal bioparameters, which served as the basis for constructing prediction models. Chilled apricot seeds collected in November were used for the experiments in March. Experiment 1 used intact seeds, while experiment 2 used overwintered larvae obtained by cracking the endocarp cover. Both larvae and intact seeds were subjected to seven constant temperatures (14.5, 18.8, 21.3, 24.0, 27.0, 30.2, and 34.3 °C). The post-diapause larvae of *E. maslovskii* developed into adults at a temperature range of 14.5–30.2 °C, and no larvae pupated at 34.3 °C. The lower temperature thresholds (LTs) for post-diapause larva and pupa and the total post-diapause period until adult emergence and until adult exit were 8.1, 8.2, 8.2, and 7.3 °C, respectively, whose thermal constants (DD) were 66.2, 180.2, 246.9, and 336.7 degree days, respectively. The distribution of *E. maslovskii* at all post-diapause stages was described using a two-parameter Weibull function. The data predicted by the model using accumulated degree days starting from January 1 did not differ by more than three days from the observed field emergence of *E. maslovskii*. Our data provide insights into the development of *E. maslovskii* after diapause. Temperature-dependent development supports the use of a degree day model to predict field emergence for pest timing control.

## 1. Introduction

The apricot seed wasp *Eurytoma maslovskii* Nikol’skaya (Hymenoptera: Eurytomidae) is a stone fruit-associated pest in Korea, China, Japan, and eastern Russia [1], which mainly attacks *Prunus* spp. (Rosales: Rosaceae), including the apricot *Prunus sibirica* L [2], *Prunus mume* Sieb. Et Zucc, *Prunus armeniaca* var. ansu Max., and peach *Prunus persica* (L.) Batsch [3]. Since its first occurrence in 1979 [4], *E. maslovskii* has become a destructive pest in the main apricot production area in Korea [3,5]. In China, the occurrence of *E. maslovskii* has been confirmed in Hebei [6], Liaoning, Chaoyang [2], Zhejiang [7], and Shandong [8]. The fruits infested by *E. maslovskii* result in the non-development of the flesh leading to a loss of apricot fruit production of 33.3–67% [5], and up to 80% of the crop being severely infested [9].

*Eurytoma maslovskii* adults emerge in spring, from early April to early May, coinciding with the fruiting periods of apricot trees [5]. The female uses her approximately 5 mm long ovipositor to deposit eggs into the soft nucleus part of the developing seed [3]. Although several eggs may be deposited within a fruit, only one larva is able to ultimately develop in each seed [5]. The entire larval stage consumes the nucleus within the endocarp for development. The larva remains inside its initially invaded seed and initiates diapause after maturation in July [3,5,7]. Pupation is estimated to begin in February of the following year [7]. Most *E. maslovskii* have one generation per year, but few complete a two-year lifecycle [6,7,8]. At present, our understanding of *E. maslovskii* development after diapause remains limited.

The incidence of *E. maslovskii* can be reduced by destroying infested fruits using a seed-crusher machine [10]. Synthetic pesticides and plant extracts have also been successfully applied to suppress the adult population; 2–3 applications during the fruiting period of apricot trees are recommended [5,11]. Recently, sex pheromones have been developed [12], providing further tools for the monitoring of these pests. In general, application timing plays an essential role in the success of this strategy. Therefore, being able to predict the spring emergence of a pest has important implications on the effectiveness of pest management.

Insects are exothermic, such that their development depends on the surrounding temperature. Each species requires a specific, consistent amount of heat accumulation, called “thermal constant” or “degree days” to reach certain life stages. Degree days (DDs) are commonly used to predict insect life cycles, and in turn, serve as time-control measures for those life cycles [13,14,15,16,17,18]. The calculation of DDs is based on the daily maximum and minimum temperatures relative to species-specific parameters, including the lower temperature threshold (LT) and upper temperature threshold (UT) [17,19]. Unfortunately, information on these thermal bioparameters is yet to be determined for *E. maslovskii*.

In this study, the influence of temperature on the development of *E. maslovskii* after a diapause break is investigated and their development rate using linear and nonlinear models is simulated. The estimated model parameters serve as the basis for the construction of reliable and accurate models for predicting the spring emergence of *E. maslovskii*.

## 2. Materials and Methods

### 2.1. Insect Source

Unfallen apricot seeds were collected from an orchard in Hwangjeon, Suncheon, Jeonnam, Korea (35.238538° N, 126.420640° E) on 23 November 2021. The seeds were brought back to the laboratory and stored in a temperature-controlled chamber (GMSR-1430; GMS Co., Ltd., Gyeonggi, Korea) at 2 °C until the following year for further experiments.

### 2.2. Temperature-Dependent Development Experiment

The experiments were initiated in early March 2022 when the diapause of *E. maslovskii* in the field was assumed to have been broken. Two experimental sets were designed to investigate the influence of seven designated constant temperatures on the developmental duration of insects.

For the first experiment (Experiment 1), groups of 50 randomly selected cold-storage seeds were kept in transparent plastic insect breeding dishes (d = 100 cm × h = 40 cm) (SPL Life Sciences, Gyeonggi, Korea). The dishes were then placed in no-light control incubators (VS-1203 P1-150-0; Vision Scientific Co., Ltd., Daejeon, Korea), with two dishes per incubator. The temperature and humidity in the incubators were recorded hourly using a HOBO Pro v2 data logger (Onset, Bourne, MA, USA); the actual temperatures were 14.5 ± 0.03, 18.8 ± 0.03, 21.3 ± 0.07, 24.0 ± 0.02, 27.0 ± 0.06, 30.2 ± 0.05, and 34.3 ± 0.02 °C in each incubator, respectively, while the humidity ranged from 40–60%. The number of adult wasps that escaped the seeds was recorded daily, and the wasps were sexed. Developmental duration was defined as the period from the start of incubation until the adults emerged from (adult exited) the seed. After no adult exits were consecutively recorded for 20 days, the seeds were broken using a walnut opener to determine the total number of overwintered larvae and the percentage of prolonged diapause larvae, if available.

The second experiment (experiment 2) was designed to investigate the development of *E. maslovskii* from diapause until adult emergence. Seeds from the refrigerator were slightly cracked using a walnut opener to take out the overwintering larvae inside. Each larva was then placed in the hole of a Combi Cryo-Box (SCB4500/81-hole type; Ecocell, Gyeonggi, Korea) with a cotton pad at the bottom. After adding forty-nine larvae to the box, the comb was covered with a transparent plastic pad. The larvae were labeled based on the number of boards in the box. Each larvae-containing box was transferred to an incubator, as described above. The pupation and adult emergence dates were recorded daily. The number of adults was counted and the individual insects were sexed. The number of individuals that died and non-pupated larvae was calculated to determine mortality and prolonged diapause. The percentages of prolonged diapause, pupation, and adult emergence were calculated by the number of overwintering larvae investigated. The developmental durations were determined within the period from the start of incubation until the emergence of the adults.

### 2.3. Selection of Model for Describing the Development Rate

We used two linear and eight commonly used nonlinear development rate models to analyze the relationship between temperature and development rate of *E. maslovskii* (Table 1). The selection of the most appropriate model was based on the following criteria:

(1) The model should comprise the desired statistical parameters to measure its standing as the model with the best fit. Here, we used the Akaike information criterion (*AIC*) (Equation (1)) and its corrected value (*AICc*) (Equation (2)) [20] for small sample size analysis to evaluate the goodness of fit of selected models.

(1) $$AIC=n\times \left[\ln\left(\frac{RSS}{n}\right)\right]+2p$$

(2) $$AICc=AIC+2p\times \frac{\left(p+1\right)}{\left(n-p-1\right)}$$

where *n* is the sample size, *p* is the number of model parameters, and *RSS* is the residual sum of squares. The model ranking is based on the *AICc*, where a lower value indicates a better fit among the candidate set of models. The Akaike weights (*w_i_*) were calculated to interpret the probability of choosing a model as the best model for the empirical data, as well as its standing relative to the second-best-fit model [21,22].

(3) $$w_{i}=\frac{\exp\left(-\frac{\Delta_{i}}{2}\right)}{\sum_{i=1}^{m}\exp\left(-\frac{\Delta_{i}}{2}\right)}$$

where *w_i_* is the weight of model *i*, Δ*i* represents the difference in the *AICc* value between model *i* and the considered best model (receiving the lowest *AICc* value), and *m* is the number of models analyzed.

(2) The models should describe the observed data accurately and have parameters that allow estimates of biological significance, including lower temperature threshold (*LT*), optimal temperature (Topt), upper temperature threshold (*UT*), and total effective thermal constant (*DD*).

**Table 1:** Models used to analyze the relationship between temperature and development rate of the post-diapause period of *Eurytoma maslovskii*.

Type	Model	Equation	Commentary	Reference
Linear	Ordinary	τ(T)=aT+b	*a* and *b* are the slope and intercept of the linear regression, respectively.	[23]
	Ikemoto	$\left(DT\right)=K+T_{min}D$	*D* is the developmental duration at temperature *T*; *K* is thermal constant (degree day) and *T_min_* is the lower temperature threshold.	[24]
Non-linear	Longan-6	$\tau \left(T\right)=\Psi \left\{e^{\rho T}-e^{\left[\rho T_{max}-\left(\frac{T_{max}-T}{\Delta T}\right)\right]}\right\}$	ψ is the developmental rate at some base temperature above the developmental threshold, *ρ* is a constant defining the rate increase to the optimal temperature, *T_max_* is the lethal temperature threshold, and Δ*T* is the temperature range over which physiological breakdown becomes the overriding influence.	[25]
	Briere 1	$\tau \left(T\right)=aT\left(T-T_{min}\right)\sqrt{\left(T_{max}-T\right)}$	*a* is an empirical constant, *T_min_* is the lower temperature threshold, and *T_max_* is the lethal temperature threshold.	[26]
	Briere 2	$\tau \left(T\right)=aT\left(T-T_{min}\right){\left(T_{max}-T\right)}^{1/d}$	*a*; *T_min_*; and *T_max_* are as in Birere-1, and *d* is an empirical constant.	[26]
	Lactin 1	$\tau \left(T\right)=e^{\rho T}-e^{\left[\rho T_{max}-\left(\frac{T_{max}-T}{\Delta T}\right)\right]}$	*ρ*, *T_max_*, and Δ*T* are the same as in Logan-6.	[27]
	Lactin 2	$\tau \left(T\right)=e^{\rho T}-e^{\left[\rho T_{max}-\left(\frac{T_{max}-T}{\Delta T}\right)\right]}+\lambda$	*ρ*; *T_max_*; and Δ*T* are the same as in the Logan-6 model, and *λ* allows the curve to intersect the abscissa at suboptimal temperatures.	[27]
	Inverse second-order polynomial	$\tau \left(T\right)=a{\left(1+bT+cT^{2}\right)}^{-1}$	*a*, *b*, and *c* are empirical constants.	[13]
	Third-order polynomial	$\tau \left(T\right)=aT^{3}+bT^{2}+cT+d$	*a*, *b*, *c*, and *d* are empirical constants.	[28]
	Simplified beta type	$\tau \left(T\right)=\rho \left(\alpha -\frac{T}{10}\right){\left(\frac{T}{10}\right)}^{\beta }$	*ρ*, *α*, and *β* are empirical constants.	[29]

### 2.4. Distribution Model of Eurytoma maslovskii Post-Diapause

Based on the results obtained from the temperature-dependent experiment, we developed a cumulative distribution model for the *E. maslovskii* developmental stages after diapause breaks, including pupation, adult emergence, and adult exit. The models were designed based on the cumulative distribution against the accumulated *DD* using the two-parameter Weibull function [30], as follows:

(4) $$F\left(x\right)=1-e^{-{\left(\frac{x}{a}\right)}^{b}}$$

where *F*(*x*) is the cumulative emergence or exit at the accumulated *DD* (*x*), and *a* and *b* are the model scale and shape parameters, respectively. The accumulated *DD* (*x*) was calculated using Equation (5):

(5) $$DD=\left(T_{a}-LT\right)\overset{n}{\underset{1}{\int }}dx$$

where *T_a_* is the average temperature in each incubator, *LT* is the lower temperature threshold estimated by the linear model, and *dx* is an integral variable representing developmental duration after *n* days of incubation.

According to our observations (as shown in the Section 4), after emerging from the pupa, the adults of *E. maslovskii* spend a certain period inside their host apricot seed before escaping, defined as post-emergence. This period should be considered when establishing prediction models, as the field appearance of *E. maslovskii* occurs after adult exit. Therefore, an adult exit model was used to simulate the spring emergence of *E. maslovskii* in the field.

### 2.5. Model Validation and Field Monitoring

The emergence of *E. maslovskii* was observed under various conditions to validate the model outcomes. On 14 March 2022, the remaining apricot seeds from the refrigerator were grouped into two groups (approximately 500 seeds per group). The first group was transferred to an insect breeding cage (l = 40 cm × w = 40 cm × h = 40 cm) maintained under laboratory room conditions. The second group was placed in a cone trap (d = 50 × h = 75 cm) and placed outdoors (semi-field) in the practice training area of Sunchon National University. A data logger HOBO Pro v2 (Onset, Bourne, MA, USA) with measurements set at hourly intervals was also placed in each cage and trapped to obtain the ambient temperature.

The emergence of *E. maslovskii* under the field conditions was observed at five different locations belonging to Jeollanam-do and Jeollabuk-do provinces in Korea from 2015–2016 and 2022, including: (1) Guryong-ri, Paldeok-myeon, Sunchang, Jeollabuk-do (35.386575° N, 127.100509° E); (2) Seolmae-ri, Gunnam-myeon, Yeonggwang, Jeollanam-do (35.238538° N, 126.420640° E); (3) Daechi-ri, Hwangjeon-myeon, Suncheon, Jeollanam-do (35.130456° N, 127.479109° E); (4) Songsan-ri, Podu-myeon, Goheung, Jeollanam-do (34.618605° N, 127.351838° E); and (5) Dosa-ri, Daap-myeon, Gwangyang, Jeollanam-do (35.078053° N, 127.715450° E). Two cone traps (as described in the semi-field experiment) were placed at each location. Dried seeds collected from the surrounding orchard were placed in traps. Adult emergence from the trap was assumed to be the field occurrence of *E. maslovskii*. The meteorological data of the locations where traps were placed were obtained from a nearby weather station (downloaded from the Weather Data Service of the Korea Meteorological Administration) (http://data.kma.go.kr (accessed on 30 June 2022)). The DD (Equation (6)) used for model input was calculated from 1 January each year based on daily maximum (*T_max_*) and minimum (*T_min_*) temperatures relative to *LT* and *UT* obtained from the linear and nonlinear model of *E. maslovskii* development rate [17,19].

(6) $$DD=\{\begin{array}{c} 0;\;if\;T_{max}<LT\\ \frac{\left(T_{max}+LT\right)}{2}-LT;\;if\;UT>T_{max}>LT>T_{min}\\ \frac{\left(T_{max}+T_{min}\right)}{2}-LT;if\;UT>T_{max}>T_{min}>LT\\ \frac{UT+T_{min}}{2}-LT;if\;T_{max}>UT>T_{min}>LT \end{array}$$

Model efficacy was evaluated based on the methods proposed by Kim and Lee (2010) and Park et al. (2014) [15,16]. Pearson’s correlation between actual observed data and model-predicted data was performed to confirm the robustness of the model outputs, of which the model comprising significantly positive values was considered to be highly robust [15]. The accuracy of the models was determined by running a one-tailed *t*-test for a null hypothesis that the mean difference in days between observed and predicted dates equals three or five days [16], with an alternative hypothesis that the mean difference is greater than three or five days. Paired data on the Julian date obtained at five cumulative flight rate points of 10, 30, 50, 70, and 90% [14] in actual observed and models predicted *E. maslovskii* adult flights were used for correlation analysis and hypothesis testing.

### 2.6. Data Analysis

After excluding outliers using a Grubbs test [31] and confirming the normality and variance homogeneity, the developmental duration data were subjected to one-way ANOVA to determine significant differences. An HSD Tukey’s test was performed for post hoc analysis. The Marquardt–Levenberg algorithm [32] was applied to find the coefficients of the independent variable(s) that resulted in the best fit between the equation and the observed data using SigmaPlot 14 (Systat Software Inc., San Jose, CA, USA). Correlation analyses and hypothesis tests were conducted using Minitab 18 (Minitab LLC, State College, PA, USA).

## 3. Results

### 3.1. Temperature-Dependent Development

Overwintering *E. maslovskii* larvae successfully developed into adults at test temperatures ranging from 14.5–30.2 °C in both experiments. Temperature significantly influenced the developmental duration of the post-diapause periods of *E. maslovskii*, including overwintered mature larvae (*df = 5*, *238*; *F = 293.04*; *p < 0.001*; Table 2), pupa (*df = 5*, *234*; *F = 2908.61*; *p < 0.001*; Table 2), and the total duration from larva until adult emergence (*df = 5*, *231*; *F = 1901.39*; *p < 0.001*; Table 2), and from larvae until adult exit (*df = 5*, *360*; *F = 2219.99*; *p < 0.001*; Table 3). The developmental duration of *E. maslovskii* at all stages after diapause break was significantly reduced in response to an increase in the tested temperatures at 14.5–27.0 °C, then gradually decreased at 30.2 °C (not different from 27 °C), until no larvae pupated at a higher temperature (34.3 °C) in the cracked seed experiment (Table 2), and no adult exited in the intact seed experiment (Table 3). At most temperatures, in both experiments (except for 18.8 °C in the cracked seed experiment), the development duration of male was significantly shorter than that of female (for the cracked seed experiment—Table 2: 14.5 °C: *df = 1*, *43*; *F = 27.24*; *p < 0.001*; 18.8 °C: *df = 1*, *38*; *F = 3.41*; *p = 0.072*; 21.3 °C:* df = 1*, *40*; *F = 12.59*, *p = 0.001*; 24.0 °C: *df = 1*, *42*; *F = 34.66*; *p < 0.001*; 27.0 °C: *df = 1*, *37*; *F = 34.52*; *p < 0.001*; 30.2 °C: *df = 1*, *25*; *F = 4.84*; *p = 0.037*. For the intact seed experiment—Table 3: 14.5 °C: *df = 1*, *71*; *F = 18.29*; *p < 0.001*; 18.8 °C: *df = 1*, *36*; *F = 48.88*; *p < 0.001*; 21.3 °C: *df = 1*, *62*; *F = 149.64*, *p < 0.001*; 24.0 °C: *df = 1*, *76*; *F = 117.26*; *p < 0.001*; 27.0 °C: *df = 1*, *67*; *F = 58.50*; *p < 0.001*; 30.2 °C: *df = 1*, *42*; *F = 24.44*; *p < 0.001*). The percentage of larvae pupated was 60.9–95.9%. A total of 58.7–93.6% adults emerged (Table 2) and 69.8–94.1% adults successfully escaped from the seed (Table 3) among the total number of examined larvae. Furthermore, the experiments showed 1.2–6.4% living larvae that did not pupate at temperatures from 14.5 to 30.2 °C, increasing to 87.9–100% at 34.3 °C. After emergence, adult *E. maslovskii* spent 4.6–7.2 days inside the seed before escaping through an exit hole variously among tested temperatures.

### 3.2. Selection of Models for Describing the Development Rate

The temperature-dependent development of post-diapause *E. maslovskii* can be accurately described using a linear model within a temperature range of 14.5–30.2 °C (*adj. r*^2^ > *0.94*). The ordinary linear model, which achieved a lower *AICc* value, fit our data better than the Ikemoto model in all cases. For the post-diapause larva, pupa, and entire post-diapause duration from larva until adult emergence, the temperature range of 14.5–30.2 °C was the most appropriate, whereas for the whole post-diapause duration until adult exit from the seed, a range of 14.5–27.0 °C achieved a better model fit (Table A1).

The nonlinear models for describing the development rates were selected from the eight commonly used candidates (Table A1). Based on *AICc* ranking, Lactin-1 and Longan-6 were found to the best fit for post-diapause larvae and the entire post-diapause period until adult emergence, while Lactin-1 and Briere-1 were the best for pupae, with the higher likelihood Akaike weight value (*w_i_*) achieved by Lactin-1. Lactin-2 and Longan-6 were the most appropriate for describing the entire duration until the adults exited the seed with a share weights of 0.734 and 0.224, respectively (Table A1).

### 3.3. Estimation of Model Parameters

Based on the ordinary linear model, the estimated LTs for the post-diapause larva, pupa, and entire post-diapause period until adult emergence with separated males and females were 8.1, 8.2, 8.2, 8.3, and 8.0 °C, respectively, with total effective thermal constants of 66.2, 180.8, 246.9, 233.6, and 256.4 DD, respectively. The estimated *LT* and *DD* were 7.3 °C and 336.7 for the entire post-diapause period until adult exit (males: 7.5 °C and 311.5, respectively; females: 7.1 °C and 361.0, respectively) (Table A2).

**Figure 1:**
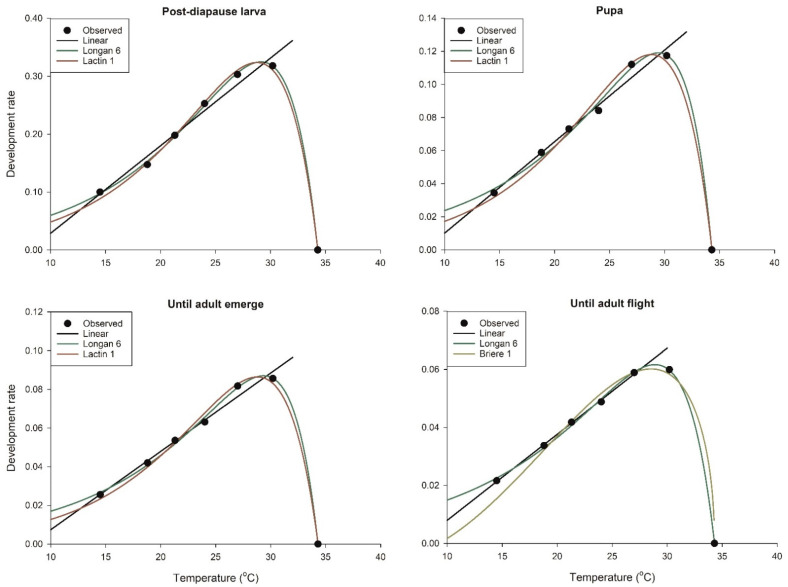
The linear and non-linear models fitted to the observed development rate of various stages of *Eurytoma maslovskii* after diapause break.

The parameter estimates for the nonlinear models are also presented in Table A2. The basal temperature (*LT*) estimated by Briere-1 was 10.5 °C for all developmental stages after the diapause break of *E. maslovskii* until the emergence of the adults. By contrast, the temperature ranged from 8.5 °C to 9.2 °C for the total period from overwintered larvae to adult exit. A large difference was observed in terms of the *UT* estimation. Lactin-1, Briere-1, and Longan-6 showed a *UT* of approximately 34.3 °C, while Lactin-2 estimated a *UT* of 36.6–39.4 °C. This conflicted with the observed data, as larvae failed to develop further at 34.3 °C. With respect to the *AICc* based-rank and model selection criteria, Lactin-1 was selected as the best model for overwintered larvae, pupae, and the entire post-diapause period until adult emergence, while Longan-6 was found to be the best fit for the entire post-diapause period until adult exit from the seed (Table A2 and Figure 1).

### 3.4. Distribution Model

**Figure 2:**
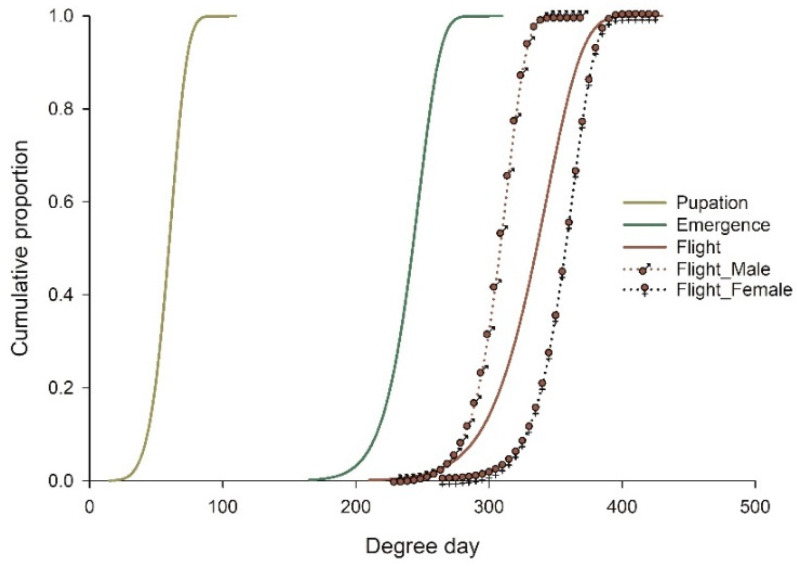
The 2-parameter Weibull distribution model for the cumulative proportion of development stages of *Eurytoma maslovskii* after diapause break by degree day.

The two-parameter Weibull function was used to describe the cumulative distribution of *E. maslovskii* after the diapause break, including pupation, adult emergence, and adult exit from the seed (flight start) (Figure 2). The high coefficient of determination achieved (*r^2^ ≥ 0.8849*) indicated that the function represented a good fit for all development stages. The parameters estimated for the models are presented in Table 4. As the flight of *E. maslovskii* adults occurred after a period of time post-emergence, the field emergence model was constructed based on adult exit data, with the *LT* and *UT* obtained from linear and nonlinear models in Table A2.

### 3.5. Field Monitoring and Model Validation

**Figure A1:**
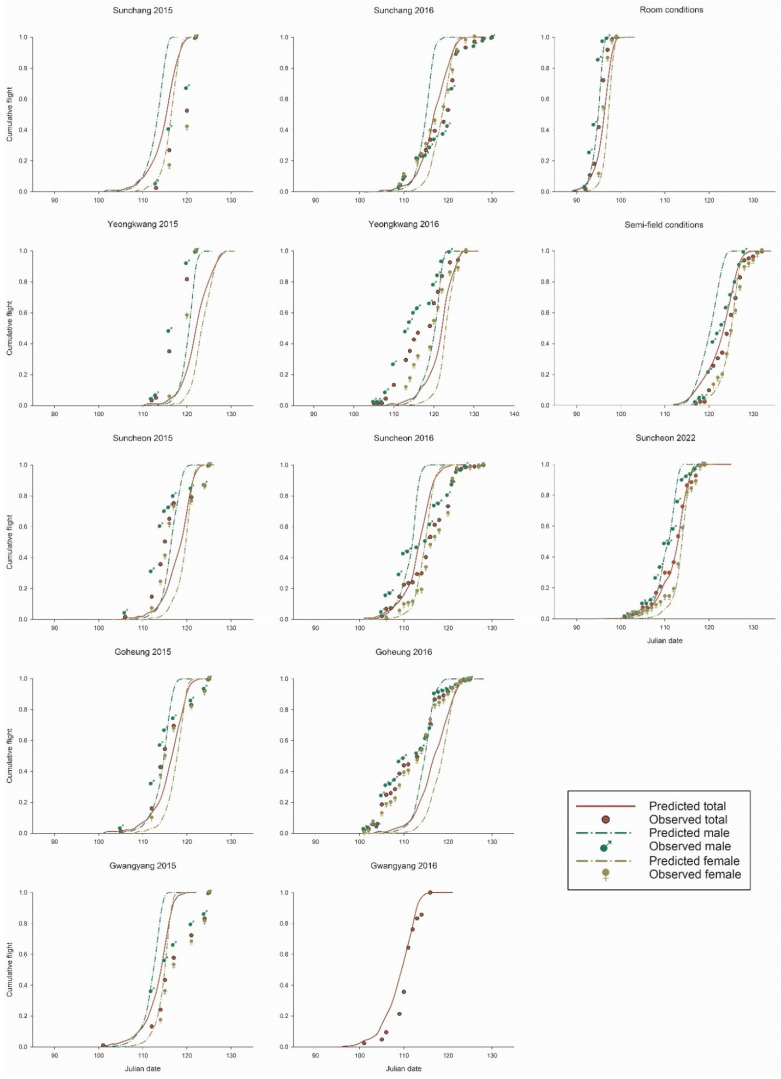
Observed and predicted cumulative flight of *Eurytoma maslovskii* at different locations and conditions in 2015–2016 and 2022.

The adult exit model performed very well under various conditions in 2015–2016 and 2022 (Table 5 and Figure A1). Based on the Julian date at cumulative flight points of 10, 30, 50, 70, and 90%, the difference in days between observed and predicted dates ranged from 0.4 to 4.6 days. As a result of the one-sample *t*-test, it was not greater than three days in 12 out of 13 surveyed points, and no location showed a difference of more than five days from the observed data. The strong correlation (*r^2^* > *0.88*) between the model prediction and actual observed data by Pearson correlation analysis indicated the high robustness of the model output.

## 4. Discussion

After the diapause break, the overwintered larvae of *E. maslovskii* remain in the larval form for some time before pupation. Before escaping the seed through an exit hole, adults spend several days developing inside the seed. We confirmed that these developments are driven by environmental temperatures. The insect developed well at a temperature range of 14.5–27.0 °C, with 83.0–93.6% of larvae successfully developing into adults (Table 2); similarly, over 80.9% of these were then able to escape from the apricot seeds in the intact seed experiment (Table 3). At 30.2 °C, which was considered higher than the optimal temperature estimated by Lactin-1, the pupation and emergence percentage dropped to 60.9% and 58.7%, respectively, and only 69.8% of adults could escape. Furthermore, exposure to a temperature of 34.3 °C led to a delay in the development of *E. maslovskii* larvae; however, this did not result in a high mortality, indicating the sensitivity of the insect to temperature.

Long life cycles are common for all orders of insects in response to environmental adversities under suboptimal conditions, including extreme weather, a lack of food or low quality of food sources, and natural enemies [33,34]. Habitat changes can stimulate certain individuals in a population to prolong their diapause [33]. For the order Hymenoptera, a dormancy period of over a year has been reported at the larval stage [35,36]. A two-year generation was also reported for *E. maslovskii* in Hebei [6], Zhejiang [7], and Shandong, China, with a larval prolonged rate of 3.89% [8]. In the present study, we also observed a similar proportion of prolonged diapause larvae at 14.5–27 °C, ranging from 2.1–4.3% (in the cracked seed experiment) and 1.2–6.4% (in the intact seed experiment), which markedly increased at higher temperatures, until all larvae postponed their further development at 34.3 °C. Although the regulation of this partly prolonged diapause is poorly understood, it may be related to metabolism. Xu et al. (2021) [36] reported that this physiological state, with the involvement of trehalose, glucose, glycerol, glycogen, lipids, and carbohydrates differs from the true annual diapause of *Cephalcia chuxiongica* Xiao (Hymenoptera: Pamphiliidae). Although metabolic processes were not investigated in the present study, it is evident that temperature was involved in regulating the prolonged behavior of *E. maslovskii*. In addition, separating larvae from their home-seed overwintered habitat may also have an impact. There was a slightly higher proportion of prolonged diapause larvae was observed in the cracked seed experiment. This was observed more clearly at 30.2 °C, wherein only 1.9% prolonged larvae occurred in the intact seeds, while this percentage increased to 21.7% in the cracked seed experiment. Further studies will be needed to clarify the relationship between these habitat changes and the metabolites responsible for prolonged diapause in *E.* *maslovskii*.

Various mathematical equations have been proposed for insect development, which can be grouped into linear and nonlinear models, wherein the selection of the model with the best fit is a crucial step in modeling practice [13,21]. Linear models are commonly used for estimating *LT* and *DD* [23,24], while nonlinear models are used to address the drawbacks of linear models, providing insights into *UT* and Topt [25,26,27]. The Akaike information criterion is commonly used in model selection for insects [13]. The models with the best fit were selected among the candidate sets using the *AICc* value [21]. In addition, it is generally important to gain further insights into the likelihood of model-given data. However, both *AIC* and *AICc* were insufficient to determine the power of a model with respect to the others in the set. The Akaike weight (*w_i_*) was calculated for this purpose. As all *w_i_* values sum to 1, they can be interpreted as probabilities [21,22]. As a result, the ordinary linear model with a temperature range of 14.5–30.2 °C was selected for post-diapause larva, pupa, and total post-diapause development until the emergence of the *E. maslovskii* adults, whereas the range of 14.5–27.0 °C was more appropriate for parameter estimation of the total post-diapause development until adult exit. For the nonlinear model, Lactin-1 fit the post-diapause larva, pupa, and total period until adult emergence with an *AIC* weight close to absolute (*w_i_* = 0.839, 0.962, and 0.908 for larvae, pupae, and larvae until adult emergence, respectively (Table A1). Lactin-2 was found to be the best model for the total post-diapause period until adult exit; however, *UT* estimated by Lactin-2 was not practical with respect to the empirically observed data. Therefore, the model with the second best fit, Longan-6, with a reasonable Akaike weight value (*w_i_* = 0.224; Table A1) was chosen in this context.

The accumulation of *DD* was successfully applied to predict the seasonal emergence of numerous insect species [13,14,15,16,17]. However, the thermal bioparameters of *E. maslovskii* were unknown prior to the present study. Previously, Wang et al. (2005) [9] proposed a method to predict the field emergence of *E. maslovskii* in Shandong from 2001 to 2003 based on a trained linear relationship between the observed emergence and accumulative average daily temperature starting from April 10, regardless of the *LT*, *UT*, and *DD* for model input. In practice, the average method may be inappropriate when the daily minimum temperature falls below the base temperature [13,17,19]. Our explanatory models, based on the thermal biology of *E. maslovskii*, may provide more reliable outcomes. In the *DD* model, determining the starting point of thermal accumulation is crucial. Similar to most other insect phenology models, January 1 was set as the biofix [14,16,17,37]. The model worked well under most of the tested conditions according to the validation processes. Furthermore, under laboratory room and semi-field conditions, where the ambient temperature was recorded directly by data loggers, the model output matched the observed data very well. Although there was a slight variation under field conditions, the predicted cumulative flight of *E. maslovskii* did not differ by more than three days from the actual observation. This difference may result from the divergence between the actual temperature in the orchards and the temperature recorded by the nearby weather station. However, in addition to our data, the use of the *DD* model can also predict the 50% cumulative emergence of *E. maslovskii* in Naju, as observed by Choi et al. [5] and by Lee et al. [3] in Suncheon, Sunchang, Yeongkwang in 2014 with a difference of two days. Although limitations remain with regard to the use of the *DD* model using temperature recorded by nearby weather stations [17], the model presented in this study showed acceptable validity in the context of a fairly dense meteorological station network in Korea.

## 5. Conclusions

This study provided insights into the development of *E. maslovskii* after a diapause break. After the diapause break, the overwintered larvae of *E. maslovskii* spent some time in the larval form prior to pupation. Adults underwent a post-emergence period before escaping the seed through an exit hole. Prolonged diapause was found to be compulsory in *E. maslovskii* larvae. The temperature-dependent development of *E. maslovskii* can be simulated using the ordinary linear model and nonlinear Lactin-1 and Longan-6 models. Estimated parameters can be used to construct phenological models to predict developmental stages in the field, providing fundamental information for decision making within pest control.

## Figures and Tables

**Table 2:** Mean (± SE) developmental duration of the post-diapause periods of *Eurytoma maslovskii* until adult emergence at various temperatures.

Temperature(°C)	n	Duration (Days)					Percentage (%)			Sex Ratio (M:F)
		Larva	Pupa	Total	Male	Female *^a^*	Prolong *^b^*	Pupation	Emergence	
14.5	49	10.0 ± 0.20 a	29.1 ± 0.19 a	39.1 ± 0.34 a	36.8 ± 0.44 a	40.0 ± 0.32 a,**	4.1	95.9	91.8	1:2.8
18.8	47	6.8 ± 0.21 b	17.0 ± 0.15 b	23.8 ± 0.29 b	23.3 ± 0.44 b	24.4 ± 0.37 b, ns	4.3	89.4	85.1	1:1.0
21.3	47	5.0 ± 0.16 c	13.7 ± 0.16 c	18.6 ± 0.22 c	18.0 ± 0.34 c	19.3 ± 0.19 c,**	2.1	91.5	89.4	1:1.0
24.0	47	4.0 ± 0.09 d	11.9 ± 0.10 d	15.8 ± 0.14 d	15.1 ± 0.16 d	16.3 ± 0.14 d,**	2.1	93.6	93.6	1:1.4
27.0	47	3.3 ± 0.07 e	8.9 ± 0.11 e	12.2 ± 0.14 e	11.3 ± 0.14 e	12.6 ± 0.13 e,**	4.3	85.1	83.0	1:2.3
30.2	46	3.1 ± 0.14 e	8.5 ± 0.10 e	11.7 ± 0.19 e	11.3 ± 0.24 e	12.1 ± 0.27 e,*	21.7	60.9	58.7	1:0.9
34.3	49	- *^c^*	-	-	-	-	100.0	-	-	
*p*-value		*<* *0.001*	*<* *0.001*	*<* *0.001*						

Means followed by the same letter within a column are not significantly different (*p* < 0.05, Tukey’s HSD test); n: number of larvae investigated (sample size); *^a^* * and ** indicate the developmental duration of females is significantly different from that of males within the same temperature at confident levels *p* < 0.05 and *p* < 0.01, respectively; ns—not significant (Tukey’s HSD test); *^b^* larvae that did not pupate but were still alive; *^c^* no development was observed.

**Table 3:** Mean (± SE) developmental duration of the post-diapause periods of *Eurytoma maslovskii* until adults exited from their hosted apricot seed at various temperatures.

Temperature(°C)	n	Duration (Days)			Percentage (%)		Post-Emergence *^d^* (Days)			Sex Ratio
		Total	Male	Female *^a^*	Prolong *^b^*	Exit	Total	Male	Female	(M:F)
14.5	81	46.3 ± 0.32 a	44.7 ± 0.55 a	47.3 ± 0.33 a,**	1.2	90.1	7.2	7.9	7.3	1:1.6
18.8	47	29.7 ± 0.33 b	27.9 ± 0.22 b	31.0 ± 0.34 b,**	6.4	80.9	5.8	4.6	6.7	1:1.4
21.3	68	23.9 ± 0.26 c	22.4 ± 0.21 c	25.8 ± 0.18 c,**	1.5	94.1	5.3	4.4	6.5	1:0.8
24.0	83	20.5 ± 0.17 d	19.0 ± 0.17 d	21.4 ± 0.14 d,**	1.2	94.0	4.6	3.9	5.1	1:1.5
27.0	74	17.0 ± 0.17 e	16.0 ± 0.20 e	17.9 ± 0.14 e,**	2.7	93.2	4.7	4.7	5.3	1:1.1
30.2	63	16.7 ± 0.24 e	15.8 ± 0.25 e	17.8 ± 0.31 e,**	1.6	69.8	5.0	4.5	5.7	1:0.8
34.3	33	- *^c^*	-	-	87.9	-				
*p*-value		*<0.001*	*<0.001*	*<0.001*						

Means followed by the same letter within a column are not significantly different at the confident level *p* < 0.05 by Tukey’s HSD test. n: number of larvae investigated (sample size); *^a^* ** indicates the developmental duration of females is significantly different from that of males within the same temperature at the confident level *p* < 0.01; *^b^* larvae that did not pupate but were still alive; *^c^* no adults exit was observed; *^d^* the developmental period of adults inside the apricot seed after emergence until they exit, calculated by the difference in mean total duration presented in Table 2 and Table 3.

**Table 4:** The two-parameter Weibull function for the cumulative distribution of *Eurytoma maslovskii* after diapause break based on accumulated degree days.

Model	Model Parameter		*r* ^2^
	*a*	*b*	
Pupation	63.2202 ± 1.1350	6.0138 ± 0.9486	*0.8849*
Adult emergence	248.2907 ± 1.4045	15.8672 ± 1.8857	*0.8928*
Adult exit	344.9728 ± 0.8859	13.5357 ± 0.6402	*0.9447*
- Male	313.8498 ± 0.8076	21.1625 ± 1.7056	*0.9316*
- Female	363.6323 ± 1.0307	21.8888 ± 1.7971	*0.9101*

*a*, *b* are the scale and shape parameters of the 2-parameter Weibull function.

**Table 5:** Accuracy of the spring model in forecasting the Julian date of cumulative flight of *Eurytoma maslovskii* adults.

Year	Location	Observed/Predicted Julian Date at Cumulative Point					Difference *^a^*	*t*-Value *^b^*		*r^2^ ^c^*
		10%	30%	50%	70%	90%		3 Days	5 Days	
2015	Sunchang	116/111	117/114	120/116	121/117	122/119	3.8 ± 0.4	2.14 ns	-3.21 ns	*0.9691*
	Yeongkwang	116/119	117/121	119/123	120/124	121/127	4.2 ± 0.5	2.45 *	-1.63 ns	*0.9778*
	Suncheon	112/114	114/117	115/119	117/120	124/122	2.8 ± 0.4	−0.53 ns	−5.88 ns	*0.8917*
	Goheung	112/112	114/115	115/117	120/119	124/120	1.6 ± 0.7	−2.06 ns	−5.01 ns	*0.9222*
	Gwangyang	112/110	115/113	117/115	121/116	124/117	3.6 ± 1.0	0.58 ns	−1.36 ns	*0.9493*
2016	Sunchang	112/113	116/116	120/117	121/119	122/121	1.4 ± 0.5	−3.14 ns	−7.06 ns	*0.9500*
	Yeongkwang	110/117	114/121	119/123	121/124	124/126	4.6 ± 1.0	1.55 ns	−0.39 ns	*0.9850*
	Suncheon	109/109	114/113	116/114	120/116	122/117	2.4 ± 0.9	−0.65 ns	−2.8 ns	*0.9911*
	Goheung	105/112	109/115	114/117	116/120	121/122	4.2 ± 1.1	1.12 ns	−0.75 ns	*0.9864*
	Gwangyang	106/105	110/108	111/110	112/112	115/113	1.2 ± 0.4	−4.81 ns	−10.16 ns	*0.9668*
2022	Room	093/094	095/096	096/097	096/097	097/098	1.0 ± 0.0	- *^d^*	-	*1.0000*
	Semi-field	120/117	122/122	125/124	126/125	128/127	1.2 ± 0.5	−3.67 ns	−7.76 ns	*0.9661*
	Suncheon	108/108	111/112	113/113	114/114	117/116	0.4 ± 0.2	−10.61 ns	−18.78 ns	*0.9828*

*^a^* Difference in days (mean ± SE) between observed and predicted Julian dates at 10, 30, 50, 70, and 90% cumulative flights; *^b^* one-sample *t*-test was applied for a null hypothesis that the mean difference in days between observed and predicted dates equals 3 or 5 days, with the alternative hypothesis means are greater than 3 or 5 days; *^c^* Pearson correlation coefficient between actual observed data and model outputs; *^d^* the differences in days are identical (equal to 1); ns: not significant; *: *p* < 0.05.

## Data Availability

The datasets generated during the current study are available from the corresponding author on reasonable request.

## Appendix A

**Table A1:** Selection of linear and non-linear models for describing the relationship between temperature and the development rate of the post diapause period of *Eurytoma maslovskii* based on the corrected Akaike Information Criterion (*AICc*), and Akaike weights (*w_i_*).

Model Type	Model Name	Temperature Range	*p*	Post-Diapause Larva					Pupa					Total Post-Diapause until Adult Emergence					Total Post-Diapause until Adult Flight			
				*Adj. r* ^2^	*AIC*	*AICc*	*w_i_*	Rank	*Adj. r* ^2^	*AIC*	*AICc*	*w_i_*	Rank	*Adj. r* ^2^	*AIC*	*AICc*	*w_i_*	Rank	*AIC*	*AICc*	*w_i_*	Rank
Linear	Ordinary	14.5–27.0	2	0.981	−48.983	−42.983	0.304	2	0.979	−53.283	−47.283	0.004	2	0.987	−58.645	−52.645	0.006	2	−72.881	−66.881	0.723	1
	Ordinary	14.5–30.2	2	0.969	−48.639	−44.639	0.696	1	0.976	−62.346	−58.346	0.996	1	0.979	−66.817	−62.817	0.994	1	−68.963	−64.963	0.277	2
	Ikemoto	14.5–27.0	2	1.000	−27.246	−21.246	0.000	4	0.987	21.821	27.821	0.000	3	0.989	23.608	29.608	0.000	3	15.930	21.930	0.000	3
	Ikemoto	14.5–30.2	2	1.000	−34.146	−30.146	0.000	3	0.984	26.454	30.454	0.000	4	0.986	29.207	33.207	0.000	4	36.539	40.539	0.000	4
Non-linear	Longan_6	14.5–34.3	4	0.998	−73.058	−53.058	0.158	2	0.990	−74.906	−54.906	0.010	3	0.996	−84.518	−64.518	0.050	2	−92.808	−72.808	0.224	2
	Briere_1	14.5–34.3	3	0.942	−52.261	−44.261	0.002	3	0.921	−64.403	−56.403	0.021	2	0.932	−72.094	−64.094	0.041	3	−76.760	−68.760	0.030	3
	Briere_2	14.5–34.3	4	0.947	−48.782	−28.782	0.000	6	0.947	−62.403	−42.403	0.000	6	0.885	−65.242	−45.242	0.000	6	−76.150	−56.150	0.000	5
	Lactin_1	14.5–34.3	3	0.995	−64.403	−56.403	0.839	1	0.983	−72.094	−64.094	0.962	1	0.989	−78.305	−70.305	0.908	1	−51.861	−43.861	0.000	8
	Lactin_2	14.5–34.3	4	0.992	−62.403	−42.403	0.001	4	0.989	−74.001	−54.001	0.006	4	0.989	−74.001	−54.001	0.000	5	−95.208	−75.208	0.743	1
	Inverse second-order polynomial	14.5–34.3	3	0.538	−33.614	−25.614	0.000	8	0.489	−47.009	−39.009	0.000	7	0.489	−47.009	−39.009	0.000	8	−55.976	−47.976	0.000	7
	Third-order polynomial	14.5–34.3	4	0.942	−48.145	−28.145	0.000	7	0.884	−57.551	−37.551	0.000	8	0.903	−62.403	−42.403	0.000	7	−70.094	−50.094	0.000	6
	Simplified beta type	14.5–34.3	3	0.943	−48.285	−40.285	0.000	5	0.919	−59.551	−51.551	0.002	5	0.928	−64.403	−56.403	0.001	4	−72.094	−64.094	0.003	4

*p*—number of model parameters.

**Table A2:** Parameters estimate for models describing the temperature-dependent development of the post-diapause periods of *Eurytoma maslovskii* from overwintered larva until adult emergence and adult exit from their hosted apricot seeds based on linear regression and non-linear regression.

Model	Parameter	Larva	Pupa	Entire Period until Adult Emergence			Entire Period until Adult Exit		
				Total	Male	Female	Total	Male	Female
Ordinary (linear)	*a*	0.0151 ± 0.0012	0.00553 ± 0.000383	0.00405 ± 0.000264	0.00428 ± 0.000377	0.00390 ± 0.000249	0.00297 ± 0.00006	0.00321 ± 0.00004	0.00277 ± 0.00007
	*b*	−0.123 ± 0.0279	−0.0452 ± 0.00890	−0.0331 ± 0.00613	−0.0354 ± 0.00874	−0.0313 ± 0.00578	−0.0217 ± 0.00133	−0.0242 ± 0.00078	−0.0196 ± 0.00145
	LT (°C)	8.1	8.2	8.2	8.3	8.0	7.3	7.5	7.1
	K (degree-day)	66.2	180.8	246.9	233.6	256.4	336.7	311.5	361.0
	*r* ^2^	*0.975*	*0.981*	*0.983*	*0.970*	*0.984*	*0.999*	*0.999*	*0.998*
Longan 6 (non-linear)	ᴪ	0.0198 ± 0.0030	0.0088 ± 0.0026	0.0061 ± 0.0013	0.0055 ± 0.0016	0.0061 ± 0.0012	0.0059 0.0009	0.0056 ± 0.0010	0.0059 ± 0.0006
	*ρ*	0.1115 ± 0.0090	0.0989 ± 0.0153	0.1021 ± 0.0114	0.1111 ± 0.0173	0.1007 ± 0.0109	0.0928 0.0087	0.1000 ± 0.0112	0.0909 ± 0.0053
	*T_max_*	34.3002 ± 0.0262	34.3000 ± 0.0574	34.3001 ± 0.0406	34.2989 ± 0.0511	34.3000 ± 0.0396	34.3008 0.0346	34.3014 ± 0.0380	34.3001 ± 0.0216
	Δ*T*	3.2438 ± 0.3315	2.7001 ± 0.5920	2.8418 ± 0.4365	3.1763 ± 0.6327	2.8156 ± 0.4204	3.0952 0.3675	3.4342 ± 0.4633	3.0839 ± 0.2263
	*r* ^2^	*0.9992*	*0.9952*	*0.9977*	*0.9968*	*0.9978*	*0.9986*	*0.9985*	*0.9995*
Briere 1 (non-linear)	*A*	0.0003 ± 0.00005	0.00009 ± 0.00002	0.00007 ± 0.00002	0.00007 ± 0.00002	0.00006 ± 0.00002	0.0005 ± 0.00009	0.0005 ± 0.00007	0.0004 ± 0.00009
	*T_max_*	34.3000 ± 1.4549	34.3000 ± 1.7050	34.3000 ± 1.5783	34.3000 ± 1.6246	34.3000 ± 1.5920	34.3000 ± 1.3359	34.3000 ± 0.9905	34.3000 ± 1.4014
	*T_min_*	10.4962 ± 1.7161	10.4981 ± 2.0106	10.5015 ± 1.8602	10.5678 ± 1.8968	10.4321 ± 1.8952	9.2334 ± 1.8743	8.5034 ± 1.5268	9.0640 ± 2.0103
	*r* ^2^	*0.9613*	*0.9471*	*0.9545*	*0.9526*	*0.9533*	*0.9620*	*0.9797*	*0.9573*
Lactin 1 (non-linear)	*ρ*	0.1800 ± 0.0037	0.1812 ± 0.0068	0.1809 ± 0.0055	0.1813 ± 0.0050	0.1802 ± 0.0056	(not fit observed data)		
	*T_max_*	34.3085 ± 0.0508	34.3136 ± 0.0933	34.3122 ± 0.0755	34.3072 ± 0.0685	34.3126 ± 0.0771			
	Δ*T*	5.5457 ± 0.1118	5.5156 ± 0.2059	5.5255 ± 0.1668	5.5126 ± 0.1515	5.5455 ± 0.1703			
	*r* ^2^	*0.9967*	*0.9888*	*0.9927*	*0.9940*	*0.9924*			
Lactin 2 (non-linear)	*Ρ*	0.0131 ± 0.0008	0.0053 ± 0.0004	0.0041 ± 0.0003	0.0044 ± 0.0004	0.0039 ± 0.0003	0.0029 ± 0.0001	0.0031 ± 0.00009	0.0027 ± 0.0001
	*T_max_*	36.6099 ± 0.4056	37.3780 ± 0.8020	37.8752 ± 0.7284	38.1434 ± 1.0169	37.9211 ± 0.7447	39.1740 ± 0.3890	39.4152 ± 0.2679	39.3332 ± 0.5015
	Δ*T*	1.8017 ± 0.3330	1.4869 ± 0.4044	1.5322 ± 0.3267	1.6873± 0.4719	1.5320 ± 0.3296	1.8680 ± 0.1591	2.0142 ± 0.1137	1.8902 ± 0.2012
	*λ*	−1.1184 ± 0.0227	−1.0473 ± 0.0100	−1.0364 ± 0.0062	−1.0407 ± 0.0100	−1.0346 ± 0.0060	−1.0215 ± 0.0025	−1.0246 ± 0.0019	−1.0194 ± 0.0029
	*r* ^2^	*0.9958*	*0.9946*	*0.9963*	*0.9917*	*0.9962*	*0.9990*	*0.9995*	*0.9984*
